# *Bacillus subtilis* YtpP and Thioredoxin A Are New Players in the Coenzyme-A-Mediated Defense Mechanism against Cellular Stress

**DOI:** 10.3390/antiox12040938

**Published:** 2023-04-15

**Authors:** Maria-Armineh Tossounian, Maria Baczynska, William Dalton, Sew Yeu Peak-Chew, Kipras Undzenas, George Korza, Valeriy Filonenko, Mark Skehel, Peter Setlow, Ivan Gout

**Affiliations:** 1 Department of Structural and Molecular Biology, University College London, London WC1E 6BT, UK; 2 MRC Laboratory of Molecular Biology, Cambridge Biomedical Campus, Cambridge CB2 0QH, UK; 3 Department of Molecular Biology and Biophysics, UConn Health, Farmington, CT 06030, USA; 4 Department of Cell Signaling, Institute of Molecular Biology and Genetics, National Academy of Sciences of Ukraine, 03680 Kyiv, Ukraine; 5 The Francis Crick Institute, 1 Midland Road, London NW1 1AT, UK

**Keywords:** coenzyme A, protein CoAlation, protein deCoAlation, mixed disulfide, oxidative stress, *Bacillus subtilis*, *Bacillus megaterium*, antioxidant

## Abstract

Coenzyme A (CoA) is an important cellular metabolite that is critical for metabolic processes and the regulation of gene expression. Recent discovery of the antioxidant function of CoA has highlighted its protective role that leads to the formation of a mixed disulfide bond with protein cysteines, which is termed protein CoAlation. To date, more than 2000 CoAlated bacterial and mammalian proteins have been identified in cellular responses to oxidative stress, with the majority being involved in metabolic pathways (60%). Studies have shown that protein CoAlation is a widespread post-translational modification which modulates the activity and conformation of the modified proteins. The induction of protein CoAlation by oxidative stress was found to be rapidly reversed after the removal of oxidizing agents from the medium of cultured cells. In this study, we developed an enzyme-linked immunosorbent assay (ELISA)-based deCoAlation assay to detect deCoAlation activity from *Bacillus subtilis* and *Bacillus megaterium* lysates. We then used a combination of ELISA-based assay and purification strategies to show that deCoAlation is an enzyme-driven mechanism. Using mass-spectrometry and deCoAlation assays, we identified *B. subtilis* YtpP (thioredoxin-like protein) and thioredoxin A (TrxA) as enzymes that can remove CoA from different substrates. With mutagenesis studies, we identified YtpP and TrxA catalytic cysteine residues and proposed a possible deCoAlation mechanism for CoAlated methionine sulfoxide reducatse A (MsrA) and peroxiredoxin 5 (PRDX5) proteins, which results in the release of both CoA and the reduced form of MsrA or PRDX5. Overall, this paper reveals the deCoAlation activity of YtpP and TrxA and opens doors to future studies on the CoA-mediated redox regulation of CoAlated proteins under various cellular stress conditions.

## 1. Introduction

Levels of reactive oxygen species (ROS) determine the fate of cells, guiding them either toward a balanced homeostatic state or an imbalanced state that leads to the expression of an array of antioxidant systems. Upon increase in cellular ROS levels above the threshold of cellular homeostasis, ROS are known to damage DNA, lipids, and proteins [1,2]. To counteract oxidative stress, cells express a variety of cellular antioxidant systems including antioxidant enzymes, reducing pathways (e.g., thioredoxin–thioredoxin reductase pathway, glutathione (GSH) pathway), and low-molecular-weight (LMW) thiols (e.g., GSH, mycothiol (MSH), bacillithiol (BSH), and coenzyme A (CoA)), among others [3,4,5,6,7,8]. These different antioxidant systems participate directly or indirectly in scavenging oxidants, rescuing macromolecules from oxidative damage and maintaining a reducing cellular environment. Protein cysteine residues are most sensitive to redox modifications during oxidative stress. Under physiological conditions (“oxidative eustress”), protein cysteine thiols become oxidized to sulfenic acid, a reversible modification [9]. However, when the level of H_2_O_2_ increases (“oxidative distress”), the sulfenic acid is further oxidized to sulfinic and sulfonic acids, which are mostly irreversible modifications [2,10]. These can, thus, interfere with the protein function and interaction with downstream partners [11].

To prevent overoxidation of proteins, cysteines can form intra- or inter-molecular disulfide bonds [1]. A protein cysteine can also form a disulfide bond with an LMW thiol, which is known as a mixed disulfide bond. GSH is an LMW thiol composed of a tripeptide, which is found in eukaryotes and Gram-negative bacteria [12,13]. Under cellular stress conditions, proteins can become glutathionylated, which prevents overoxidation to sulfonic acid, loss of protein function, and subsequent degradation [14]. Following glutathionylation, glutaredoxins (Grx) reduce the GSH–protein disulfide bond, which leads to the transfer of GSH onto Grx and the release of the reduced protein. Another GSH molecule is then capable of reducing the GSH–Grx mixed disulfide bond, which results in the formation of a glutathione disulfide (GSSG) and the release of the reduced Grx. Glutathione disulfide reductase than reduces GSSG while using NADPH as its final electron donor [15,16].

Coenzyme A (CoA) is a key cellular metabolite that is composed of a 3′-phosphorylated ADP-moiety and a pantetheine tail, which has a reactive thiol group at its end. Unlike GSH, CoA is found in all organisms [17]. In recent years, its role in the cellular antioxidant response has been shown [5,6]. Studies on mammalian and bacterial cells subjected to different oxidizing conditions have shown significant increases in covalent protein modification by CoA. The sites of protein CoAlation were identified by using anti-CoA antibodies combined with liquid chromatography tandem mass-spectrometry (LC-MS/MS) analysis [18,19,20]. To date, over 2000 mammalian and bacterial CoAlated proteins have been identified and grouped in the CoAlome [21]. Structural studies and bioinformatics analyses of known CoAlated proteins have revealed that CoA is a flexible molecule that is capable of adapting its conformation to access the micro-environment of protein cysteine residues [21]. In vitro and in cell studies of different proteins (e.g., nucleoside diphosphate kinase 1 (NME1), peroxiredoxin 5 (PRDX5), aurora kinase A (AurA), glyceraldehyde-3-phosphate dehydrogenase (GAPDH), transcription factor AgrA) have shown that CoAlation is a widespread and reversible modification that leads to changes in protein catalytic activity, in addition to local structural changes surrounding the site of CoAlation [19,22,23,24,25,26,27]. Immunohistochemistry analysis of post-mortem brain samples of various neurodegenerative pathologies with anti-CoA antibodies showed significant increases in anti-CoA immunoreactivity when compared to matched control samples [28]. The immunoreactive signal was notably increased in brain samples from individuals who had Alzheimer’s disease, and the CoAlation of tau protein was shown both in vitro and in cells. Studies of bacterial and mammalian cells that were subjected to different oxidative stress conditions have also revealed significant increases in protein CoAlation. Moreover, upon removal of the oxidizing agent, the recovery of cells from oxidative stress was associated with a rapid decrease in protein CoAlation to the level in untreated cells [19,20]. This indicates that recovered cells undergo a process of deCoAlation, which involves the removal of CoA from modified proteins.

We used *Bacillus subtilis* as a model organism to determine whether protein deCoAlation is enzyme-driven, followed by the identification and characterization of enzymes which drive this process. *Bacillus* species are Gram-positive bacteria, which have a wide range of physiological characteristics that allow them to survive and live in different environments. Its most-studied member is *B. subtilis*, which is a model organism for other pathogenic members of the *Bacillus* species, such as *Bacillus anthracis* (causative agent of anthrax) and *Bacillus cereus* (causative agent of food poisoning). These bacteria have CoA, cysteine, and bacillithiol as their cellular LMW thiols. In the presence of oxidative stress, *B. subtilis* proteins are protected from oxidative damage through CoAlation and bacillithiolation [29,30].

In this study, we showed that protein deCoAlation is an enzyme-driven mechanism by using a combination of ELISA-based deCoAlation assays and different purification strategies. We selected two protein candidates: YtpP (thioredoxin-like protein) and thioredoxin A (TrxA) from mass-spectrometry analysis of purified fractions which contained the highest deCoAlation activity. Both candidates showed deCoAlation activity toward CoAlated MsrA and PRDX5. By generating single cysteine to serine mutants, we identified Cys28 (YtpP) and Cys29 (TrxA) as the major nucleophilic cysteine residues required for the deCoAlation of their substrates. Overall, we have established an ELISA-based method which can detect deCoAlation activity from lysates and have identified two *B. subtilis* enzymes, YtpP and TrxA, which can deCoAlate proteins by using catalytic cysteine residues.

## 2. Materials and Methods

### 2.1. ELISA-Based deCoAlation Assay Using B. subtilis and B. megaterium Lysates

To detect protein CoAlation from cell lysates, we developed an enzyme-linked immunosorbent assay (ELISA)-based deCoAlation methodology. ELISA plate wells (F16 MaxiSorp loose Nunc-Immuno Module—Thermo Scientific, Horsham, UK) were coated with 1.5 ng/µL (75 µL) of CoAlated glyceraldehyde 3-phosphate dehydrogenase (CoA-GAPDH; sample preparation is described below) overnight (O/N) at 4 °C. The wells were then washed 5× with 200 µL of wash solution (1X Phosphate-Buffered Saline pH 7.4 (1X PBS), 0.05% Tween20 solution, 4 min) and blocked with 5% albumin in 1X PBS buffer for 2 h at room temperature (RT). The wells were washed with wash solution and incubated with either *B. megaterium* or *B. subtilis* lysates (150 and 300 ng/µL) (75 µL) for 30 min at 37 °C. The solutions in the wells were then discarded, and the wells were washed 5× with wash solution. Primary mouse anti-CoA monoclonal antibody (75 µL; 1:6000 in 5% albumin) [31] was added to each well, and the plate was incubated O/N at 4 °C. The wells were then washed 5× with wash solution. The secondary goat anti-mouse horseradish peroxidase (HRP)-conjugated IgG antibody (75 µL; 1:5000 in 5% albumin—Promega, Southampton, UK) was added, and the plate was incubated for 1 h at RT in the dark.

The wells were washed 5× with wash solution, and the HRP-substrate solution (1 mg 3,3′,5,5′-tetramethylbenzidine (TMB—Aldrich, Gillingham, UK), 1 mL dimethyl sulfoxide (DMSO) stock, 9 mL of 50 mM phosphate-citrate buffer pH 5.0, 1.78 mM hydrogen peroxide (H_2_O_2_)) was added for 7 min at RT (dark). Upon addition of the substrate, the solution turned either dark or light blue depending on the amount of CoAlated protein present. The darker the color of the solution, the more CoAlated protein is present within the well. The reaction was stopped by adding 1 M sulfuric acid (H_2_SO_4_), which turned the solution dark or light yellow, depending on the amount of CoAlated protein present. The darker the color, the more CoAlated protein present. The absorbance at 450 nm was measured by using a CLARIOstar Plus spectrophotometer (BMG LABTECH). Several controls were used: (1) sample buffers (lysis buffer, 1X PBS, and purification buffer) were added to coated/blocked wells, where a high absorbance at 450 nm was detected; (2) 10 mM dithiothreitol (DTT—BioChemica, Darmstadt, Germany), a reducing agent, was added to coated/blocked wells, where a low absorbance at 450 nm was observed due to the reduction of the CoA–protein mixed disulfide bond; (3) a non-coated well was incubated with the sample buffer, and the absorbance that was recorded (background noise) was removed from the sample absorbance values. Three independent replicates for each condition were measured, and the results were presented in a bar graph by using GraphPad Prism 8 (version 8.3.1).

### 2.2. Growth, Harvesting, and Lysis of B. megaterium and B. subtilis Cells

*B. megaterium* and *B. subtilis* cultures (1 L/cell type) were grown in nutrient broth 3 (NB3) and Luria Broth (Miller) media, respectively, at 37 °C until OD_600_ reached mid-exponential. The cells were then harvested via centrifugation for 20 min at 6200× *g* at 4 °C in a Beckman JLA 8.1000 rotor. The pellet was resuspended in 50 mM Tris pH 8.0, 25 mM NaCl, and 1x PIC (cOmplete^TM^ Mini Protease Inhibitor Cocktail—Roche, Welwyn Garden City, UK). To lyse the cells, sonication was performed on ice by using Soniprep150 (15 cycles—15 s pulse ON/20 s pulse OFF). Following sonication, the samples were centrifuged for 30 min at 39,000× *g* at 4 °C in a Beckman JA25.50 rotor. The cell lysates were then filtered by using a 0.2 μm Minisart^®^ filter (Sartorius, Epson, UK). The proteins within the lysate were quantified with the Pierce™ Bicinchoninic acid (BCA) Protein Assay (Thermo Scientific) by using the CLARIOstar Plus spectrophotometer (CLARIOstar^®^ software version 5.61).

### 2.3. Endogenous Purification of Enzymes with deCoAlation Activity from B. megaterium Lysates

The filtered *B. megaterium* cell lysate was applied to an anion exchange chromatography (AEX) column (HiTrap™ Q HP—Cytiva, Amersham, UK) connected to an ÄKTA Start protein purification system (Cytiva). The unbound proteins were washed off the column by passing 5 column volumes (CV) of wash buffer (50 mM Tris pH 8.0 and 25 mM NaCl) through the column. Bound proteins were eluted with an increasing NaCl gradient elution of 0–50% (15 CV) followed by 50–100% (6 CV) by using a combination of wash buffer and elution buffer (50 mM Tris pH 8.0 and 1 M NaCl). The protein concentration within each fraction was measured by using the Pierce™ BCA Protein Assay (Thermo Scientific), a protein quantification assay. Where needed, due to high salt concentration, the samples were diluted.

The deCoAlation activity of each protein fraction collected from the anion exchange chromatography purification step was measured by using the ELISA-based deCoAlation assay. The CoA–GAPDH (1.5 ng/μL) coated wells were incubated with 150 ng/μL of each purified fraction at 37 °C for 30 min. As a negative control, only buffer (purification buffer or 1X PBS) was added to the coated well, and as a positive control, 10 mM DTT was added to the coated well. To remove background from wells, non-coated but blocked (5% albumin) wells were incubated with buffer. The detailed ELISA-based deCoAlation assay is described above. The results are shown in a bar graph generated via GraphPad Prism 8 (version 8.3.1).

The anion exchange chromatography purification fraction pools (A1, A2, and A3) that corresponded to the three independent deCoAlation activities were concentrated, incubated with 10 mM DTT, and then injected on a size-exclusion chromatography (SEC) column (Superdex™ 200 Increase 10/300 GL—Cytiva) equilibrated with 50 mM Tris pH 8.0 and 300 mM NaCl. The column was connected to an ÄKTA Pure purification system (Cytiva). The deCoAlation activity of each fraction was measured by using the ELISA-based deCoAlation assay (as described above). The purity of the SEC fractions (S1, S2, and S3) showing deCoAlation activity were assessed by using SDS-PAGE gel (Instant Blue staining). These samples were then analyzed via liquid chromatography with tandem mass spectrometry (LC-MS/MS) to determine the identity of the proteins.

### 2.4. Mass-Spectrometry Analysis of Purified Fractions That Showed deCoAlation Activity

The size-exclusion chromatography fractions (S1, S2, and S3) with high deCoAlation activity were analyzed via LC-MS/MS to identify potential candidates with protein deCoAlation activity. LC-MS/MS data analysis was performed as described previously [22]. The mass spectrometry proteomics data of S1, S2, and S3 have been deposited to the ProteomeXchange Consortium via the PRIDE [32,33,34] partner repository with the dataset identifier PXD041347.

### 2.5. Selection of Candidate Proteins with Possible deCoAlation Activity

From the list of the LC-MS/MS identified proteins, further selection criteria were placed to identify potential enzyme candidates with deCoAlation activity. Analogous to glutaredoxins, the selection criteria include (1) oxidoreductases, which have a conserved thioredoxin fold; (2) proteins that have cysteine residues; and (3) proteins within the molecular mass range of 10–17 kDa (based on the SEC molecular mass prediction).

To select for the oxidoreductases, the list of *B. bombysepticus *(*B. megaterium* LC-MS/MS reference set organism) oxidoreductases were retrieved from National Center for Biotechnology Information (NCBI) and UniProt. Both lists were joined, and duplicates were removed. Excel was used to compare the LC-MS/MS analysis output with the retrieved list of oxidoreductases. Following the selection of oxidoreductases, we then further reduced the list of proteins by selecting for proteins that have cysteine residues and are within a specific molecular mass range (10–17 kDa).

### 2.6. B. subtilis YtpP and TrxA Wild-Type Gene Design and Site-Directed Mutagenesis

*B. subtilis* YtpP (*B. subtilis *subsp.* subtilis* strain 168—UniProt: O34357)-pET28a(+) (wild type (WT) and cysteine mutants (C28S, C31S and C62S)) and WT TrxA (*B. subtilis *subsp.* subtilis* strain 168—UniProt: P14949)-pET28a(+) plasmids were designed and purchased from Twist Bioscience. The genes contain an N-terminal 6xHis-tag. The plasmids were electroporated in *Escherichia coli* BLR(DE3) cells by using a Gene Pulser™ (BIO-RAD). Small-scale expression tests were used to determine the optimal protein expression conditions.

*B. subtilis* TrxA cysteine mutants (C29S and C32S) were generated by using QuickChange^TM^ site-directed mutagenesis protocols (Stratagene). The primers used were TrxA C29S, Fw 5′-GCACCATGGTCTGGCCCATGCAAGATG-3′, and Rv 5′-CATCTTGCAT GGGCCAGACCATGGTGC-3′; TrxA C32S, Fw 5′-GCA CCA TGG TGT GGC CCA TCC AAG ATG-3′, and Rv 5′-CATCTTGGATGGGCCACACCATGGTGC-3′. Mutagenesis was confirmed by sequencing the plasmids. The plasmids were then electroporated in BLR(DE3) cells.

### 2.7. Expression and Purification of WT and Cysteine Mutants of YtpP and TrxA

BLR(DE3) cells containing the YtpP-pET28a(+) (WT, C28S, C31S, and C62S) or the TrxA-pET28a(+) (WT, C29S, and C32) plasmids were grown at 37 °C in LB medium (Miller) until OD_600_ reached 0.8. Cells were induced with 0.5 mM isopropyl β-D-1-thiogalactopyranoside (IPTG) and incubated O/N at 30 °C. Centrifugation (20 min at 6200× *g* at 4 °C—Beckman JLA 8.1000 rotor) was used to harvest cells, and the pellets were resuspended in a resuspension buffer (50 mM Tris pH 8.0, 0.7 M NaCl, 5 mM imidazole (Millipore, Watford, UK), 1 mM beta-mercaptoethanol (Sigma-Aldrich, Gillingham, UK), 1x PIC (cOmplete^TM^ Mini Protease Inhibitor Cocktail—Roche, Welwyn Garden City, UK), 50 μg/mL deoxyribonuclease I (DNase I Bovine pancreas—Sigma) and 10 mM MgCl_2_). Following resuspension, the cells were sonicated (as described above) and centrifuged (30 min at 39,000× *g* at 4 °C using Beckman JA25.50 rotor). The cell lysate was then filtered, and in-batch incubated with Talon affinity resin (Generon) equilibrated in binding buffer (50 mM Tris pH 8.0, 0.7 M NaCl, 5 mM imidazole, and 1 mM beta-mercaptoethanol) for 1 h at 4 °C (with gentle rotation). The beads were packed in an empty Xk16/20 column (GE Healthcare), and the column was connected to the ÄKTA Start purification system (Cytiva). The unbound proteins were washed out of the column by passing through binding buffer until A_280nm_ reached zero. A gradient elution (0–100% elution buffer) was performed to elute bound proteins by using the elution buffer (50 mM Tris pH 8.0, 0.7 M NaCl, 250 mM imidazole, and 1 mM beta-mercaptoethanol). The fractions that showed an increase in A_280nm_ were collected, and a Bradford assay (Thermo Fisher Sceintific, Waltham, MA, USA) was used to measure the protein concentration. The sample purity was assessed by using SDS-PAGE.

Another purification step was used to further purify the protein sample. The concentrated protein was injected into a size-exclusion chromatography column (HiLoad™ 16/600 Superdex™ 200 pg—Cytiva) equilibrated with 50 mM Tris pH 8.0, 0.5 M NaCl, and 5 mM DTT. The fractions which showed an increase in A_280nm_ were collected, and the Bradford assay was used to measure the protein concentration. Finally, the purity was assessed by using SDS-PAGE, and the pure sample fractions were combined and stored with 5% glycerol and 5 mM DTT at −20 °C.

### 2.8. Expression and Purification of GAPDH, MsrA, and PRDX5

The expression and purification of *Staphylococcus aureus* GAPDH, *Corynebacterium diphtheriae* MsrA, and human PRDX5 are as described above with minor modifications. The BLR(DE3) cells were grown in LB medium for 3 h at 37 °C, followed by induction with 0.5 mM IPTG. For MsrA, the cells were grown in Terrific broth (TB) medium (Sigma-Aldrich, Gillingham, UK) instead of LB.

### 2.9. CoAlation of GAPDH, MsrA, and PRDX5

Prior to protein CoAlation, the proteins were incubated with 20 mM DTT for 30 min at RT. The samples were then injected onto Superdex™ 200 Increase 10/300 GL column (Cytiva) connected to an ÄKTA Pure purification system and equilibrated with 1X PBS buffer. The protein samples were concentrated by using Vivaspin^®^ 6 concentrators (10 kDa cut-off), and their concentration was measured by using Nanodrop. For in vitro CoAlation assays, the proteins were incubated with CoA sodium salt hydrate (Sigma-Aldrich) and hydrogen peroxide (H_2_O_2_) at a molar ratio of 1:3:10 (protein:CoA:H_2_O_2_). The samples were incubated at RT for 30 min. The reaction was stopped by injecting the sample onto the Superdex™ 200 Increase 10/300 GL (Cytiva) column equilibrated with 1X PBS buffer. The peak fractions were then collected, and protein CoAlation was confirmed by using the anti-CoA Western Blot (WB—see the section below). The CoAlated protein fractions were collected and stored at −20 °C.

### 2.10. Protein deCoAlation Assay Using anti-CoA Western Blot

Prior to the deCoAlation assay, YtpP and TrxA (WT and mutants) were incubated with 20 mM DTT for 30 min at RT. The samples were then injected onto a HiTrap^TM^ desalting column (Cytiva) connected to an ÄKTA Start purification system and equilibrated with 1X PBS buffer. YtpP and TrxA (WT and mutants) were incubated with CoA–MsrA (1:16 ratio of TrxA/YtpP to CoA–MsrA, respectively) or CoA–PRDX5 (1:30 ratio of TrxA/YtpP to CoA–PRDX5, respectively) for 30 min at 37 °C. The reaction was stopped by the addition of 28 mM N-ethylmaleimide (NEM; 10 min, RT, dark). Non-reducing SDS-PAGE loading dye was then added. The samples were boiled and centrifuged, and then ~1–2 μg was loaded onto an SDS-PAGE gel (mPAGE^TM^ 4–20% Bis-Tris precast gel—Millipore).

Following SDS-PAGE, anti-CoA western blot (WB) was performed. The Bio-Rad Transfer-Blot Turbo system was used to transfer proteins from the SDS-PAGE gel onto the methanol-activated membrane (Immunobilon^®^—FL transfer membrane, Millipore). The membrane was then blocked for 25 min at RT (gentle shaking) by using the blocking buffer (LI-COR Intercept^®^ Blocking buffer—TBS) and then incubated with mouse anti-CoA primary antibody (1/6000 dilution in Intercept antibody dilution solution TBS-T20, REF) for 1.5 h at RT or O/N at 4 °C. The membrane was washed with 1X Tris-Buffer Saline solution supplemented with 0.05% Tween20 (TBSt), and the secondary goat anti-mouse Alexa-Fluor680 antibody (1/10,000 dilution in Intercept antibody dilution solution TBS-T20) was added. The membrane was incubated for 30 min and then washed with 1X TBSt, and 1X TBS. LiCor Odyssey CLX and the Image Studio Lite Software (LI-COR Biosciences) were used to determine the CoAlation signal at 700 nm.

### 2.11. Detection of CoA Release following deCoAlation by Using High-Performance Liquid Chromatography

To monitor the release of CoA following protein deCoAlation by WT YtpP or TrxA, Shimadzu high-performance liquid chromatography (HPLC) was used. The release of CoA was monitored at A_254nm_ [35] by using an isocratic elution with 150 mM sodium phosphate pH 6.4 and 9% methanol solution. To determine the elution time of CoA, varying concentrations (1, 2.5, 5, 7.5, and 10 μM) of CoA standards were injected. The deCoAlation reaction mixture consisted of CoA–MsrA or CoA–PRDX5 (6 μM), which were incubated with excess WT YtpP or TrxA (60 μM) for 1 h at 37 °C. Afterward, the samples were diluted 5X in 150 mM sodium phosphate pH 6.4 with 9% methanol solution and centrifuged to remove precipitates, and the supernatant was injected onto the Kinetex-C18 column (Phenomenex). As a positive control, the CoAlated proteins were incubated with 0.5 mM DTT for 1 h at 37 °C. To remove residual CoA signal (background), we analyzed CoA–MsrA, CoA–PRDX5, YtpP, TrxA, and DTT as control samples.

## 3. Results

### 3.1. Protein deCoAlation Activity Is Detected in B. megaterium and B. subtilis Lysates by Using an ELISA-Based deCoAlation Assay

**Figure 1:**
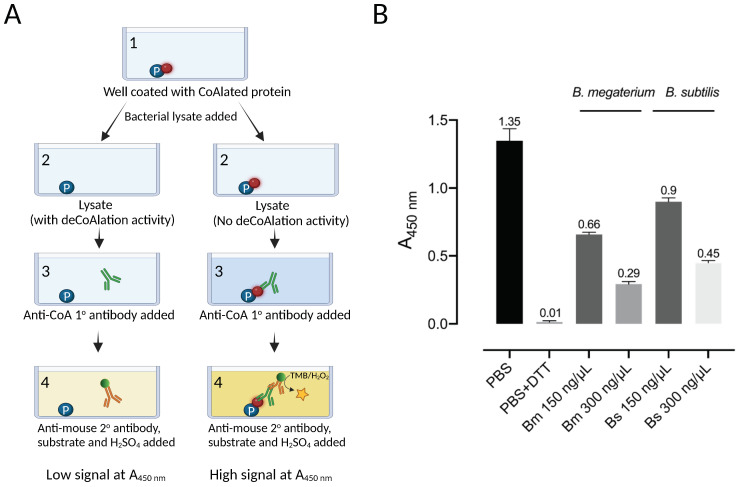
*B. megaterium* and *B. subtilis* lysates show deCoAlation activity. (**A**) Schematic diagram of ELISA-based deCoAlation assay. The plate is coated with CoAlated proteins (CoA–P). The protein is shown in a blue circle and CoA in a red circle. In the presence of lysates containing deCoAlation activity, CoA is released, while in lysates where deCoAlation activity is absent, CoA remains covalently bound to the protein. Then, the primary anti-CoA antibody (green) and secondary HRP-conjugated anti-mouse antibody (orange) are added. In the presence of TMB and H_2_O_2_, the color of the solution turns dark blue if CoA is still bound to the protein. H_2_SO_4_ is then added to stop the reaction, and the color of the solution turns dark yellow. This results in a higher signal at A_450nm._ The figure was generated by using BioRender. (**B**) The bar graph summarizes the ELISA-based deCoAlation assay using coated CoAlated-GAPDH as a substrate. In the presence of either *B. megaterium* or *B. subtilis* lysates (150 and 300 ng/μL), concentration-dependent deCoAlation activity is observed. As a negative control, 1X PBS or lysis buffer was added to the coated/blocked wells, which showed a high A_450nm_ signal. As a positive control, 10 mM DTT was added to the coated wells, where we observed loss of A_450nm_ signal, indicating deCoAlation of the coated CoA–GAPDH. The values above the bar graphs correspond to the average recorded A_450nm_ for each condition. Data from 3 independent replicate experiments are presented, and the bar graph was generated by using GraphPad Prism 8 (version 8.3.1).

An ELISA-based protein deCoAlation assay was developed to study the deCoAlation activity in *B. megaterium* and *B. subtilis* lysates (Figure 1A). Briefly, the ELISA wells were coated with a single type of CoAlated protein (*S. aureus* CoA–GAPDH), and different concentrations (150 and 300 ng/μL) of *B. megaterium* or *B. subtilis* lysates were added into each well. Mouse anti-CoA antibody was used to detect the presence of CoAlated proteins within wells, and an HRP-conjugated goat anti-mouse secondary antibody was used to detect the absorbance at 450 nm (A_450nm_) upon the addition of HRP substrates (TMB and H_2_O_2_). The reaction was stopped by H_2_SO_4_. In the presence of varying concentrations of *B. megaterium* and *B. subtilis* lysates, a decrease in signal at A_450nm_ was observed, showing the presence of protein deCoAlation activity within both lysates (Figure 1B). The decrease in A_450nm_ was slightly higher in *B. megaterium* lysate compared to the *B. subtilis* lysate. As a negative control, 1X PBS or lysis buffer was added to the coated/blocked-well, where high signal was recorded due to the detection of CoA on the CoAlated protein (Figure 1B). As a positive control, 10 mM DTT was added to a coated/blocked well, where a significant decrease in signal at A_450nm_ was recorded due to the reduction of the mixed disulfide bond between CoA and the coated CoAlated-protein.

### 3.2. Purification of Endogenous Enzymes with deCoAlation activity from B. megaterium Lysates

**Figure 2:**
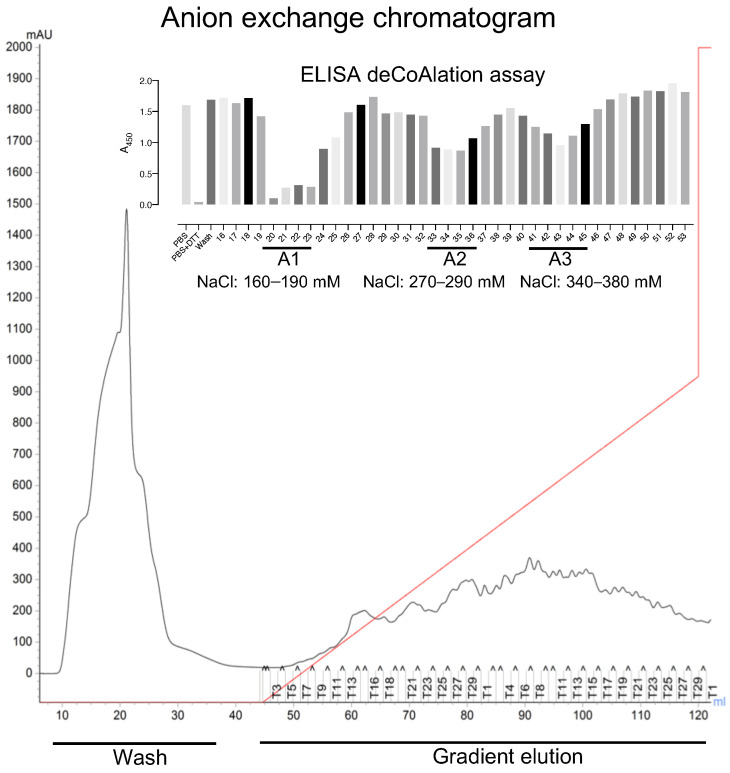
Three peaks of deCoAlation activities are observed from anion-exchange chromatography purification of *B. megaterium* lysate. The AEX chromatogram is shown. The *B. megaterium* lysate was applied to the column, and the unbound proteins were washed out of the column (Wash step). The bound proteins were eluted by using a NaCl gradient (red line) (Gradient elution). The absorbance (A_280nm_) of the bound and unbound proteins are indicated by a black line. The figure insert shows the ELISA-based deCoAlation assay bar graph, where three deCoAlation activities are observed (A1, A2, and A3). The concentration of NaCl for each fraction with deCoAlation activity is shown. The bar graph was generated by using GraphPad Prism8 (version 8.3.1).

To determine whether protein deCoAlation is enzyme-catalyzed, we used a combination of protein purification techniques and ELISA-based deCoAlation assays to purify endogenous protein candidates from lysates. We selected the *B. megaterium* lysate for further purification studies, as it showed slightly higher deCoAlation activity than the *B. subtilis* lysate (Figure 1B). To purify endogenous enzymes that deCoAlate proteins, the filtered *B. megaterium* lysate was injected onto an AEX chromatography column (HiTrap™ Q HP—Cytiva). The unbound proteins were washed from the column, and the bound proteins were eluted with an increasing NaCl gradient (Figure 2).

ELISA was used to detect deCoAlation activity within the purified fractions (bound and unbound proteins) (Figure 2—Insert). The samples (150 ng/μL) were added onto coated (1.5 ng/μL CoA–GAPDH) and blocked (5%-albumin) ELISA wells and incubated for 30 min at 37 °C. The wells were then incubated with primary anti-CoA antibody, followed by secondary HRP-conjugated anti-mouse antibodies. HRP substrates and stop solution were added, and A_450nm_ was measured by using a spectrophotometer (Figure 1A). Three purified fraction pools showed deCoAlation activity: fractions 20–23 (A1), 33–35 (A2), and 40–45 (A3), which were eluted at NaCl concentrations of 160–190 mM, 270–290 mM, and 340–380 mM, respectively (Figure 2—Insert). The strongest deCoAlation activity was observed in the first fraction pool (Figure 2 Insert—A1). To determine whether high NaCl concentration would interfere with the ELISA assay, the coated and non-coated wells were incubated with the purification buffer with high NaCl concentrations, and no effect on A_450nm_ was observed.

**Figure 3:**
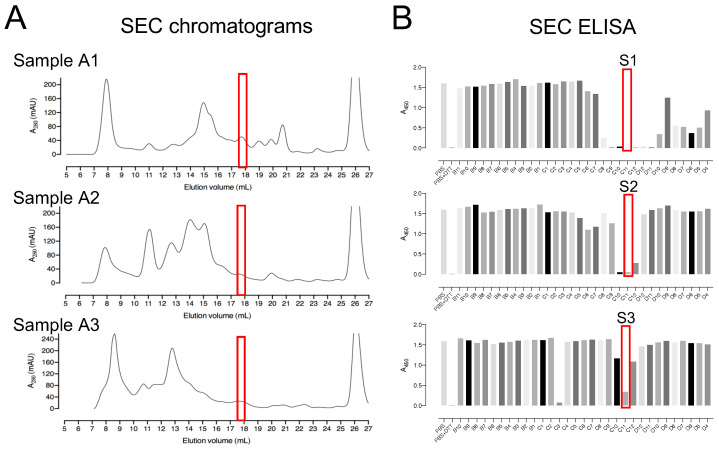
Size-exclusion chromatography as a second step for the purification of enzymes with deCoAlation activity. (**A**) Size-exclusion chromatograms of A1, A2, and A3 anion exchange fraction pools. The black line represents the A_280nm_, and the peak eluted at 25–28 mL elution volume represents DTT. (**B**) The red box indicates the fractions showing the highest deCoAlation activity (S1, S2, and S3) measured by ELISA. The bar graph was generated by using GraphPad Prism 8 (version 8.3.1).

**Figure 4:**
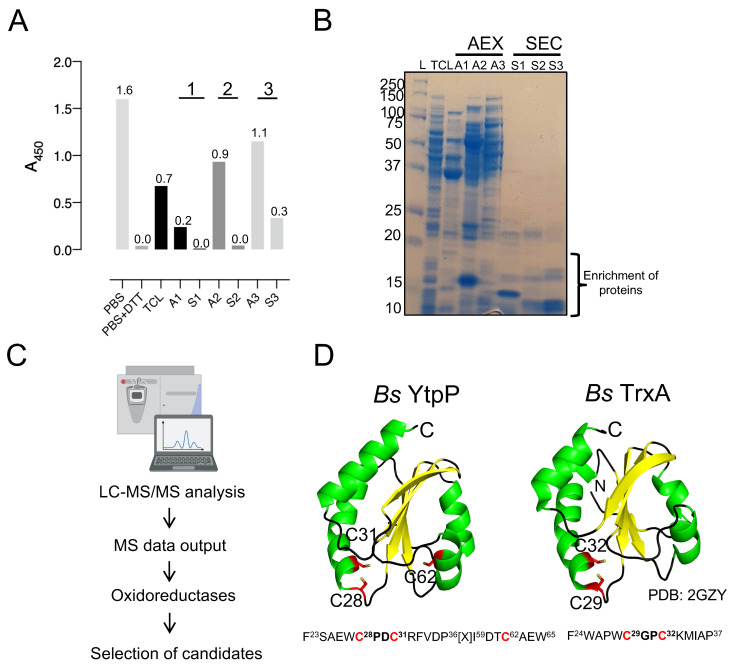
Identification of protein candidates with protein deCoAlation activity. (**A**) The bar graph shows the enrichment of deCoAlation activity after each purification step: AEX chromatography fractions (A1, A2, and A3) and SEC (S1, S2, and S3). For each sample, A_450 nm_ is indicated on top of each bar graph. The graph was generated by GraphPad Prism8 (version 8.3.1). For the A1, A2, and A3 absorbances, it is a representation of the average absorbance of the pooled fractions. (**B**) The purity of the AEX (A1, A2, and A3) and SEC (S1, S2, and S3) purification fractions are shown on an SDS-PAGE gel. The gel is stained with InstantBlue. (**C**) The purified fractions were analyzed via LC-MS/MS, and candidates were selected based on 3 criteria: belong to the oxidoreductase family, have cysteine residues, and have a specific molecular mass. Based on these criteria, YtpP and TrxA were selected as candidates. (**D**) The AlphaFold structure of *B. subtilis* YtpP is shown in cartoon form [37,38]. It has three cysteine residues (C28, C31, and C62) and a Trx-fold with a CXXC motif. The *B. subtilis* TrxA (PDB: 2GZY) structure is shown in cartoon form. The two structures have a CXXC motif and a Trx-fold. The Cys residues are shown in red and labeled. The sequences surrounding the CXXC motifs are shown below the structure.

The three pooled sample fractions (A1, A2, and A3) were separately incubated with 10 mM DTT to keep the proteins in a reduced state. The samples were subjected to SEC by using a Superdex™ 200 Increase 10/300 GL column (Figure 3A). ELISA was used to detect deCoAlation activity within the purified fractions (Figure 3B). The samples with the highest deCoAlation activity (Figure 3—samples S1, S2, and S3—red box) have a similar molecular mass (10–17 kDa—based on SEC protein standards—Figure S1). From the total cell lysate (TCL) to AEX chromatography to SEC, there is an enrichment in the deCoAlation activity (Figure 4A). It is important to note that 150 ng/µL of TCL, AEX, and SEC samples was added to each ELISA well for comparative activity assessment.

### 3.3. Mass Spectrometry Identification of Candidate Enzymes That deCoAlate

The purity of the total cell lysate (TLC) and the fractions of anion exchange chromatography (A1, A2, and A3) and size-exclusion chromatography (S1, S2, and S3) with the highest protein deCoAlation activities were assessed by using SDS-PAGE (Figure 4B). Compared to the TCL, impurities were removed after each purification step. Samples S1, S2, and S3 show the enrichment of proteins which have a molecular mass of ~10–15 kDa (Figure 4B). LC-MS/MS was used to identify the proteins within each fraction (S1, S2, and S3) (Figure 4C). Data are available via ProteomeXchange with identifier PXD041347. To select a protein with the potential of having deCoAlation activity, three criteria were used: (1) belong to the oxidoreductase family (and have a conserved thioredoxin fold); (2) have a cysteine residue(s); and (3) have a molecular mass range of 10–17 kDa, based on the molecular mass prediction of S1, S2, and S3 samples by using size-exclusion chromatography protein standards (Figure S1). The first two criteria are based on analogy to the well-characterized glutaredoxin and mycoredoxin. Two potential candidates, YtpP and thioredoxin A (TrxA), were selected in this study and further characterized for their protein deCoAlation activity. LC-MS/MS analysis showed that YtpP (~12 kDa) was present in the purified SEC fractions S2 and S3, while TrxA (~11 kDa) was in S1. The SDS-PAGE gel analysis revealed an enrichment of proteins at ~10–15 kDa, which correspond to the molecular masses of YtpP and TrxA (Figure 4B).

As the genome of the *B. subtilis* is well studied, we chose the orthologs of the *B. megaterium* protein candidates for further analysis. *B. subtilis* YtpP (Uniprot: O34357) is a thioredoxin-like protein with a molecular mass of 12.6 kDa and 107 amino acids, which contains 3 cysteine residues (Cys^28^, Cys^31^, and Cys^62^). It has a signature thioredoxin fold and a CXXC motif with unique amino acid residues flanking the two cysteines (CPDC—Figure 4D). The AlphaFold structure of *Bs* YtpP shows that the C^28^PDC^31^ motif is located 10–12 Å away from Cys^62^. The second candidate, *B. subtilis* TrxA (UniProt: P14949), has a thioredoxin fold, a CXXC motif (CGPC), and two cysteine residues (Cys^29^ and Cys^32^) (Figure 4D). Thioredoxin orthologs have been shown to dethiolate proteins (remove LMW thiols). Therefore, TrxA is a good candidate to test whether it can deCoAlate proteins. Both TrxA and YtpP belong to the oxidoreductase family and are members of the SigA and the Spx regulons [36], which regulate genes involved in cellular stress response.

### 3.4. B. subtilis YtpP and TrxA deCoAlate CoA–PRDX5 and CoA–MsrA

**Figure 5:**
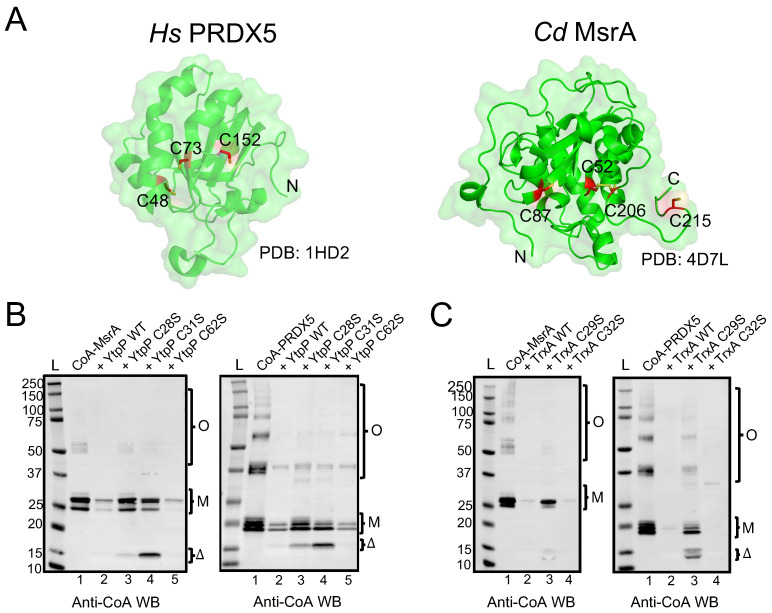
*B. subtilis* YtpP and TrxA deCoAlate CoA–MsrA and CoA–PRDX5. (**A**) Three-dimensional structures (green cartoon) of *Hs* PRDX5 (PDB: 1HD2) and *Cd* MsrA (PDB: 4D7L) are shown. The cysteine residues are shown in red sticks either buried within the structure or solvent-exposed. Anti-CoA WBs show that both *B. subtilis* (**B**) YtpP and (**C**) TrxA are capable of deCoAlating CoA–MsrA and CoA–PRDX5. “L” represents the protein ladder (kDa); Lane 1 represents the CoAlated protein (MsrA or PRDX5); Lane 2 represents the CoAlated protein in the presence of either YtpP or TrxA; Lane 3 represents the CoAlated protein in the presence of YtpP C28S or Trx C29S; Lane 4 represents the CoAlated protein in the presence of YtpP C31S or TrxA C32S; Lane 5 represents the CoAlated protein with YtpP C62S. The WBs shown are from 3 independent replicate experiments, and an equal amount of sample was loaded into each well. “M” represents the monomeric form of CoA–MsrA and CoA–PRDX5; “O” represents the oligomeric forms of CoA–MsrA and CoA–PRDX5; “Δ” represents either CoAlated YtpP C28S or YtpP C31S, or CoAlated TrxA C29S.

To determine whether YtpP and TrxA can deCoAlate proteins, the recombinant *B. subtilis* enzymes were expressed and purified (Figure S2), and their deCoAlation activity was assessed with anti-CoA WB by using different CoAlated substrates: *C. diphtheriae* methionine sulfoxide reductase A (CoA–MsrA; 26 kDa) and human peroxiredoxin 5 (CoA–PRDX5; 18 kDa) (Figure 5A). PRDX5 and orthologs of Msr are found within the identified protein CoAlome (bacterial and mammalian CoAlated protein dataset) [19,20,21] and, therefore, were chosen for this study. To CoAlate the substrates, the recombinant MsrA and PRDX5 were purified, reduced (Figure S3—blue line), and in vitro CoAlated in the presence of H_2_O_2_ and CoA. Following CoAlation, excess CoA and H_2_O_2_ were removed by SEC (Figure S3), and anti-CoA WB was used to confirm the CoAlation of both proteins (Figure 5B—Lanes 1).

Both CoAlated MsrA and PRDX5 have monomeric and oligomeric CoAlated bands (Figure 5B—Lanes 1). In addition, the monomeric bands have multiple CoAlated bands for CoA–MsrA (~ 26 kDa) and for CoA–PRDX5 (~20 kDa), which could be due to the CoAlation of different oxidized states of MsrA and PRDX5 (Figure 5B,C—Lanes 1). These oxidized states are generated in the presence of H_2_O_2_ during the in vitro CoAlation of proteins, which facilitates the formation of intra- and inter-molecular disulfide bonds, in addition to CoAlation. To examine the deCoAlation activity of YtpP and TrxA, anti-CoA WB was used (Figure 5B,C). In the presence of a molar excess of WT YtpP, almost complete deCoAlation of CoA–MsrA and CoA–PRDX5 occurs (Figure 5B—Lanes 2), showing that YtpP can reduce the mixed disulfide bond between CoA and a substrate protein. Similarly, in the presence of an excess of TrxA, we observed complete deCoAlation of both MsrA and PRDX5 (Figure 5C—Lanes 2).

### 3.5. Cys^28^ and Cys^31^ Are the Catalytic Cysteines of YtpP

To understand the protein deCoAlation mechanism, we compared the deCoAlation activities of the WT YtpP with its three single Cys-to-Ser mutants (C28S, C31S, and C62S). CoAlated substrates, CoA–MsrA or CoA–PRDX5, were incubated with WT or single Cys-to-Ser mutants of YtpP, and the samples were analyzed by using anti-CoA WB (Figure 5B). The mutation of Cys^62^, which is located 10–12 Å away from the YtpP CXXC motif (Figure 4D), does not influence the deCoAlation of both substrates, which excludes it from the catalytic deCoAlation mechanism of YtpP (Figure 5B—Lanes 5). The mutation of Cys^31^ (C31S mutant—Figure 5B Left—Lane 4) shows deCoAlation of MsrA monomeric upper (partial deCoAlation) and lower (~complete deCoAlation) bands and the MsrA oligomeric bands (complete deCoAlation). On the other hand, in the presence of YtpP C31S mutant, almost complete deCoAlation of CoA–PRDX5 monomeric and oligomeric forms was observed (Figure 5B Right—Lane 4). In both samples, a minor CoAlated YtpP C31S band (~15 kDa) appeared, which indicates the formation of the CoAlated Cys^28^ following its nucleophilic attack on the CoAlated substrate.

On the other hand, mutation of YtpP Cys^28^ (C28S mutant) shows minor deCoAlation of CoA–MsrA monomeric and oligomeric bands (Figure 5B Left—Lane 3). Incubation of YtpP C28S mutant with CoA–PRDX5 shows complete deCoAlation of the oligomeric bands and minor deCoAlation of the monomeric bands (Figure 5B Right—Lane 3). This shows that depending on the substrate and localization of the CoAlated cysteine residue, Cys^31^ may initiate the deCoAlation of proteins. Overall, these results show that both Cys^28^ and Cys^31^ are required for the efficient deCoAlation of MsrA (Figure 5B Left—Lanes 1–4), while the presence of only Cys^28^ was observed to efficiently deCoAlate PRDX5 (using C31S mutant—Figure 5B Right—Lane 4).

### 3.6. TrxA Cys^29^ and Cys^32^ Are the Catalytic Cysteine Residues Required for Protein deCoAlation

To identify the catalytic cysteine residues of TrxA that are involved in the protein deCoAlation mechanism, we generated two single Cys-to-Ser mutants (TrxA C29S and C32S mutants). Unlike YtpP single mutants, in the absence of Cys^32^ (TrxA C32S mutant), which is the second Cys residue within the TrxA CXXC motif, complete deCoAlation of both CoA–MsrA and CoA–PRDX5 is observed (Figure 5C—Lanes 4). This shows that Cys^29^ plays an important nucleophilic role in deCoAlating substrate proteins. The TrxA C29S mutant still deCoAlates both substrates, but less efficiently compared to the C32S mutant. Interestingly, following deCoAlation of CoA–PRDX5, we observe CoAlated TrxA C29S band ~14 kDa. Overall, these results indicate that Cys^29^ can efficiently deCoAlate substrates; however, Cys^32^ can also deCoAlate efficiently, depending on the substrate and localization of the CoAlated Cys.

### 3.7. CoA Is Released following deCoAlation by YtpP and TrxA

**Figure 6:**
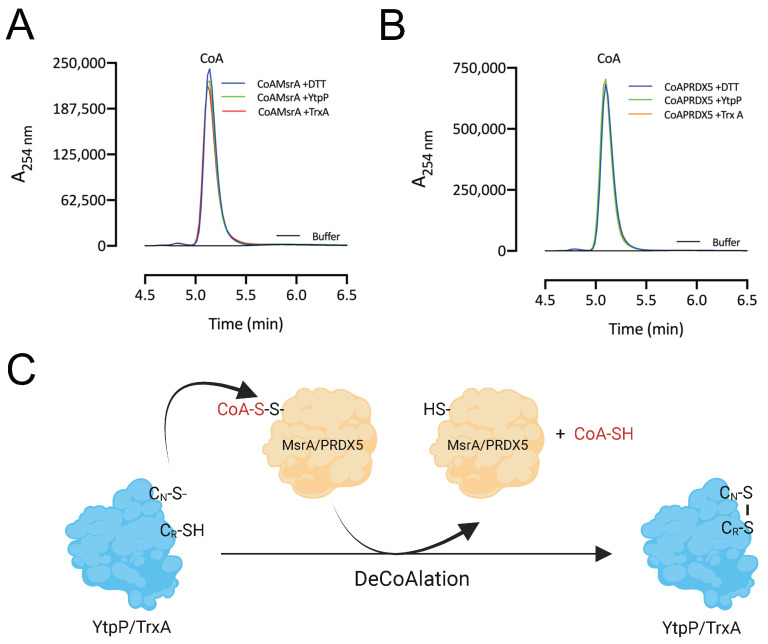
DeCoAlation of CoA–MsrA and CoA–PRDX5 by YtpP and TrxA. Release of CoA was monitored following the deCoAlation of (**A**) CoA–MsrA and (**B**) CoA–PRDX5, in the presence of YtpP and TrxA. DeCoAlation of CoA–MsrA and CoA–PRDX5 with DTT was used as a positive control. In the presence of YtpP and TrxA, CoA chromatograms are similar to that of CoA–MsrA/CoA–PRDX5 incubated with DTT. The graphs were generated by using GraphPad Prism 8 (version 8.3.1). (**C**) Proposed potential deCoAlation mechanism of MsrA and PRDX5 by YtpP and TrxA. C_N_ and C_R_ represent the nucleophilic and resolving Cys residues, respectively.

After deCoAlation of both MsrA and PRDX5, wild-type YtpP and TrxA show an absence of CoAlation (Figure 5B,C—Lanes 2). This indicates that CoA is released after substrate deCoAlation, possibly through intramolecular disulfide bond formation between the nucleophilic and resolving cysteine residues of YtpP and TrxA. To confirm this observation, we performed deCoAlation of CoA–MsrA and CoA–PRDX5 in the presence of YtpP or TrxA (Figure 6A,B) and monitored CoA release at A_254 nm_ by using HPLC [35]. To determine the elution time of CoA, known concentrations of CoA standards were injected onto a Kinetex-C18 column, and the elution volume of CoA was observed to be at ~5.2 min (Figure S4). As a positive control, CoAlated proteins were incubated with DTT, and the release of CoA was also monitored. Following deCoAlation of both substrates by YtpP and TrxA, CoA release was monitored. The heights of all CoA peaks from incubations with YtpP or TrxA were almost identical to the CoA peak from the incubation of the CoAlated substrates (MsrA and PRDX5) with DTT (Figure 6A,B and Table S1). This indicates that YtpP and TrxA catalyze complete protein deCoAlation, as observed in the anti-CoA WBs (Figure 5B,C), and CoA is released together with the reduced substrates, while the deCoAlating enzymes are themselves oxidized.

Overall, based on our results, we propose the following mechanism of deCoAlation of MsrA and PRDX5 by YtpP or TrxA (Figure 6C): the nucleophilic cysteine residue of YtpP (Cys^28^)/TrxA (Cys^29^) deCoAlates CoA–MsrA/CoA–PRDX5 by performing a nucleophilic attack on the sulfur of CoA. Then, depending on the substrate, the resolving cysteine of YtpP (Cys^31^)/TrxA (Cys^32^) attacks the CoAlated nucleophilic cysteine of YtpP/TrxA, which results in the release of CoA and the formation of an intramolecular disulfide bond (Figure 6C). Important to note is that the second cysteine residue of both YtpP and TrxA are also capable of deCoAlating, possibly depending on the localization and …“the type of”… amino acid residues surrounding the site of CoAlation on substrate proteins.

## 4. Discussion and Conclusions

Coenzyme A is an essential cofactor found in all organisms. The presence of its terminal cysteine-derived thiol group expands its function from cellular metabolism toward contributing to the cellular antioxidant system. Over the past years, studies have focused on understanding the antioxidant role of CoA during cellular stress [5,6]. An increase in protein CoAlation has been observed in different organisms in the presence of oxidative and metabolic stress, showing the antioxidant role of CoA. In particular, protein CoAlation has been shown to increase in *B. subtilis* and *B. megaterium* cells subjected to H_2_O_2_ stress, NaOCl stress, and glucose deprivation [19,29]. This paper is the first study to expand our knowledge on protein deCoAlation, which is crucial for the recovery of the catalytic activity of proteins.

Protein *S*-thiolation is well studied and causes modulation in the catalytic activity of proteins [3,5,6,12,39,40,41,42]. If the site of *S*-thiolation is within or in the vicinity of the active site of the protein, it is more likely to impact the catalytic activity of the protein. For example, CoAlation, glutathionylation, mycothiolation, and bacillithiolation of GAPDH analogs inhibit the catalytic activity of the protein [19,25,43,44,45]. To restore the reduced and active form of proteins, organism-specific antioxidant enzymes, such as glutaredoxin (Grx; reduces GSH–protein disulfide), mycoredoxin (Mrx; reduces MSH–protein disulfide), and bacilliredoxin (Brx; reduces BSH–protein disulfide) play an important role by catalyzing the reduction of the LMW thiol–protein disulfide bond [3,5,6,12,39,40,41,42]. These enzymes are well studied; however, the identity and mechanism of enzymes that deCoAlate proteins have not been reported. In this study, we identified *B. subtilis* YtpP and TrxA, as enzymes that deCoAlate different substrates (MsrA and PRDX5). Both enzymes are oxidoreductases and members of the SigA and Spx regulons, which regulate genes involved in cellular stress response [36,46,47]. *B. subtilis* contains TrxA and different Trx-like proteins including YtpP [48,49,50]. Compared to YtpP, the function and structure of TrxA are well studied, and it has been reported to be an essential protein for the growth and viability of *B. subtilis*, and it is induced by various stress conditions [49].

By generating cysteine mutants of YtpP and TrxA, we were able to identify their catalytic cysteine residues (nucleophilic and resolving cysteines) and the mechanism of deCoAlation (Figure 5B,C and Figure 6C). The main catalytic function of TrxA is the reduction of intramolecular disulfide bonds [51]. However, it has also been reported to reduce sulfenic acids and LMW thiol–protein mixed disulfide bonds. *B. subtilis* TrxA structural analysis revealed that the N-terminal cysteine of the CXXC motif has a lower *pKa* (thiolate form) than the C-terminal cysteine, which renders it more nucleophilic. The N-terminal nucleophilic cysteine attacks the intramolecular disulfide bond on the substrate, resulting in an intermediate TrxA–substrate complex that contains an intermolecular disulfide bond. Then, the C-terminal cysteine attacks this disulfide bond, resulting in the release of the reduced substrate and the formation of an intramolecular disulfide bond between the cysteine residues of the TrxA CXXC motif. This is the mechanism used by Trx enzymes, where the oxidized form of Trx is then recycled by NADPH-dependent TrxR [51,52,53,54]. In our study, both TrxA and YtpP deCoAlate proteins, which results in the release of the reduced substrate and the formation of an intramolecular disulfide bond on TrxA/YtpP. This mechanism differs from what has been reported for protein deglutathionylation by glutaredoxin, where GSH is transferred onto Grx, and the reduced protein is released. Another GSH molecule is then capable of reducing the GSH–Grx disulfide bond, which results in the release of GSSG and the reduced Grx. NADPH-dependent glutathione disulfide reductase than reduces GSSG [15,16]. Future studies might shed light on enzymes which could use a similar mechanism as Grx to deCoAlate proteins. This mechanism might use a CoA disulfide reductase (CoADR) to reduce oxidized CoA (CoASSCoA). CoADR has been reported in a few organisms (e.g., *S. aureus*). A study on *C. glutamicum* mycothiol peroxidase (Mpx) has shown that aside from mycoredoxin1, Trx can also demycothiolate Mpx but with a lower efficiency than that of Mrx1 [55]. Future kinetic studies could determine the rate of deCoAlation of proteins by TrxA/YtpP and determine whether these enzymes are capable of efficiently deCoAlating proteins.

Coenzyme A is a bulky but flexible molecule capable of adapting its conformation to form a mixed disulfide bond with solvent-exposed or buried protein cysteines [21]. A detailed study of CoAlated protein structures revealed that CoA forms different stabilization interactions within the CoAlation site. The ADP-moiety of CoA is mainly stabilized by hydrophobic residues and/or π–π stacking interactions with aromatic residues, while the pantetheine tail is stabilized by alternating polar–non-polar residues. The phosphate groups of CoA are stabilized by positively charged amino acids or water molecules [21]. To understand how TrxA or YtpP could recognize the CoAlated substrate, we further studied the known *B. subtilis* TrxA structure in complex with a substrate (heterodimer) and the homodimeric disulfide bonded form of TrxA (Figure S5A) [52,56]. TrxA has a central hydrophobic core with four β-strands, which are surrounded by alpha-helices (Figure S5A,B). It contains a conserved hydrophobic pocket close to its N-terminal cysteine residue, which binds hydrophobic residues located on the substrate molecule. The structural study of the homodimeric *B. subtilis* TrxA C32S mutant showed that this cysteine mutant is more redox-sensitive and tends to form an intermolecular disulfide bond. The Trp28 of the first TrxA subunit, which is located next to the N-terminal cysteine, is stabilized by the hydrophobic pocket (Ala26, Trp28, Val57, Ala64, Val69, and Ile72) located on the second TrxA subunit (Figure S5A,B). This pocket is shown to be important for the stabilization interactions between the two TrxA subunits. The backbones of Met70 and Ser71 pack against Trp28 for further stabilization [52]. This would allow the two nucleophilic cysteine residues to move closer and form a disulfide bond. Another structure of TrxA complexed with a substrate arsenate reductase (ArsC) showed that both molecules interact mainly via hydrophobic interactions, and notably, the side chain of TrxA Met70 and Met91 of ArsC is inserted into the other subunit, further stabilizing their interaction [56]. This interaction is also observed in human a Trx–Ref1 complex structural study [57].

As the overall 3D structures of TrxA and YtpP are similar, we compared the residues located within the vicinity of the catalytic site (Figure S5B,C). The hydrophobic pocket, which is close to the nucleophilic cysteine residue, is also present within the AlphaFold-generated YtpP structure (Figure S5C). These residues, particularly Trp28 for TrxA and Trp27 for YtpP, could play a role in the stabilization of the ADP-moiety of CoA, which is present on the substrate protein. This would lead to structural changes that allow the TrxA/YtpP nucleophilic cysteine residues to perform an attack on the mixed disulfide bond between CoA and the substrate protein. This results in the release of the reduced substrate and the transfer of CoA onto the TrxA/YtpP nucleophilic cysteine. Future structural and mutagenesis studies could open doors to understanding the interactions formed between TrxA/YtpP and the CoAlated protein substrate.

*B. subtilis* is a model organism for other pathogenic members of the *Bacillus* species (*B. anthracis* and *B. cereus*). It is a Gram-positive bacterium which has been reported to contain cysteine, CoA, and bacillithiol (BSH) as LMW thiols [42,58]. Upon cellular stress, approximately 206 proteins have been identified to be CoAlated, and 6 have been identified to be bacillithiolated [29,30]. Increases in CoAlation during *B. megaterium* sporulation has been reported, and several CoAlated proteins were identified in *B. subtilis* spores [22]. These studies show the protective role of CoA during cellular stress and highlight the importance of enzymes that deCoAlate proteins and restore their activity. Although we have focused on bacterial enzymes that deCoAlate proteins, it is important to note that approximately 1170 CoAlated mammalian proteins were identified from rat tissues, and HEK293/Pank1β cells were subjected to oxidative stress [21]. To restore the function of these CoAlated proteins, enzymes that deCoAlate would play an important role. The majority of the CoAlated mammalian proteins were involved in cellular metabolism (60%) and stress response (8%) [21]. These include enzymes facilitating glycolysis, the Krebs cycle, amino acid metabolism, and the electron transport chain, among others. Several key players of the antioxidant response are within the identified CoAlated proteins, including catalase, thioredoxins, peroxidases, thiol reductases, and Keap1, an inhibitor of the transcription factor Nrf2, which is a key regulator of the cellular antioxidant response. Therefore, understanding the molecular mechanisms of the antioxidant function of CoA would be of great interest for future studies. In a preliminary study, we used the ELISA-based deCoAlation assay to test whether deCoAlation activity is observed in mammalian cell/tissue lysates (HEK293/Pank1β cell lysates and rat liver tissue lysates). Interestingly, we observed deCoAlation activity in both lysates (unpublished data). These preliminary studies open doors for the identification of mammalian enzymes with deCoAlation activity which have a crucial role in restoring the function of CoAlated proteins and the level of reduced CoA. Overall, in this paper, we used *B. subtilis* as a model organism to other pathogenic bacteria to understand its cellular defense mechanisms. We identified two enzymes (TrxA and YtpP), which are capable of deCoAlating different substrates (MsrA and PRDX5) and determined their catalytic mechanism. This paper opens doors to future studies which aim to understand the CoA-mediated redox regulation of important cellular proteins. This knowledge in turn can be used to design experiments that weaken the antioxidant system of pathogenic bacteria by targeting its antioxidant defense mechanisms.

## Data Availability

Data is contained within the article or Supplementary Material.

## Supplementary Materials

The following supporting information can be downloaded at: https://www.mdpi.com/article/10.3390/antiox12040938/s1. Figure S1: Prediction of protein molecular mass by using gel filtration protein standards; Figure S2. Purification of recombinant *Bs* WT YtpP and TrxA; Figure S3. CoAlation of PRDX5 and MsrA in the presence of H_2_O_2_, Figure S4. HPLC chromatograms of CoA standards; Figure S5. Structural analysis of *B. subtilis* TrxA and YtpP; Table S1. CoA is released during deCoAlation of CoA–MsrA and CoA–PRDX5.
