# Supplementary material for: Bacillus subtilis YtpP and Thioredoxin A Are New Players in the Coenzyme-A-Mediated Defense Mechanism against Cellular Stress
Source: Antioxidants (Basel). 2023 Apr 15;12(4):938. doi: 10.3390/antiox12040938 (PMC10136147; doi:10.3390/antiox12040938)
Supplement: Supplementary file 1 [file antioxidants-12-00938-s001.zip › antioxidants-2257475-supplementary.pdf]

Supplementary materials

Supplementary Figures

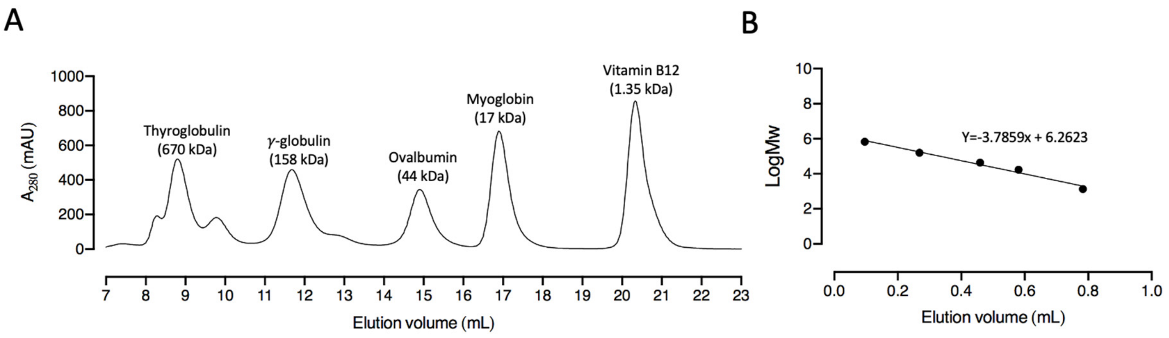

Figure S1. Prediction of protein molecular mass using gel filtration protein standards. (A) SEC chromatogram of protein standards with known molecular mass (Mw). **(B)** LogMw versus elution volume of the gel filtration protein standards are shown. The linear line equation is shown and is used to predict the molecular mass of proteins with known elution volume, but unknown molecular mass.

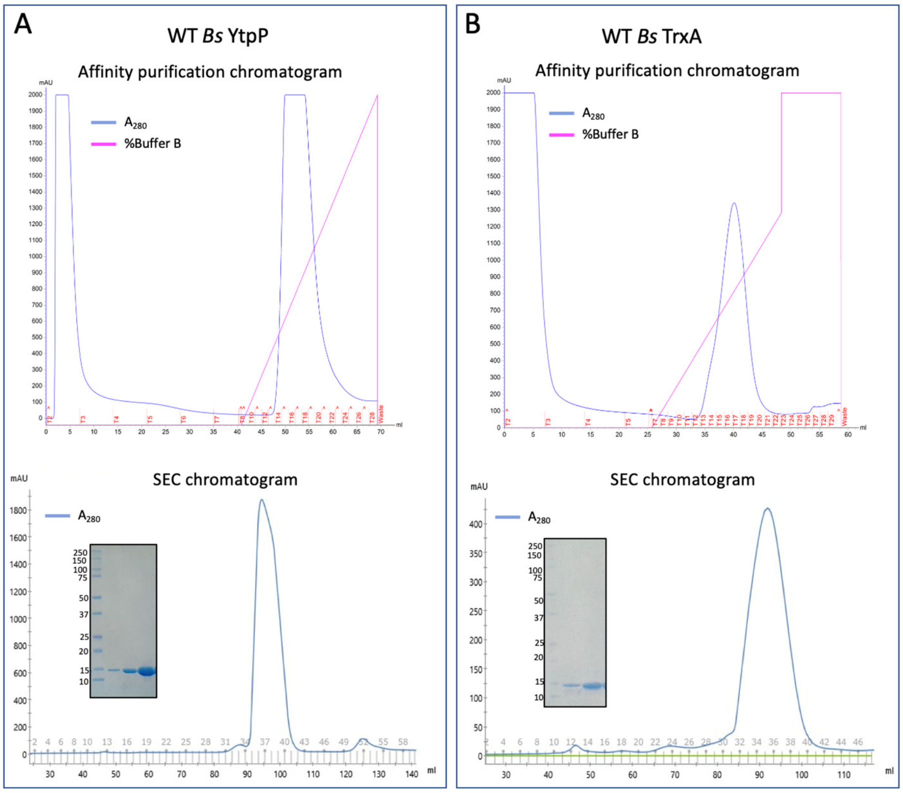

Figure S2. Purification of recombinant *Bs* WT YtpP and TrxA. The affinity purification chromatograms and the SEC chromatograms of WT **(A)** YtpP and **(B)** TrxA are shown. The blue line represents the absorbance at 280 nm and the pink line represents the percentage of the elution buffer (% Buffer B). The purity of the proteins is assessed by SDS-PAGE gel, which is stained by InstantBlue. Around 2-4 µg of protein was loaded on the SDS-PAGE gel.

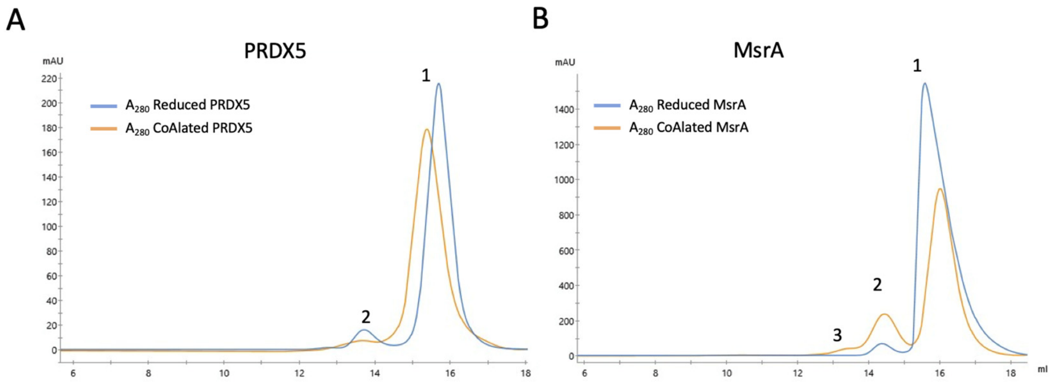

Figure S3. CoAlation of PRDX5 and MsrA in the presence of H_2O_2. Size exclusion chromatography chromatograms of the reduced (blue lines) and CoAlated (orange lines) forms of **(A)** PRDX5 and **(B)** MsrA are shown. **(A)** Following PRDX5 reduction, only peak 1 (blue line) was selected for further CoAlation in the presence of CoA and H_2O_2. Peak 1 (orange line) was collected for further deCoAlation studies. **(B)** Similarly, reduced MsrA (peak 1 – blue line) was CoAlated. Three peaks were observed (orange line), but only peak 1 was chosen for further deCoAlation studies.

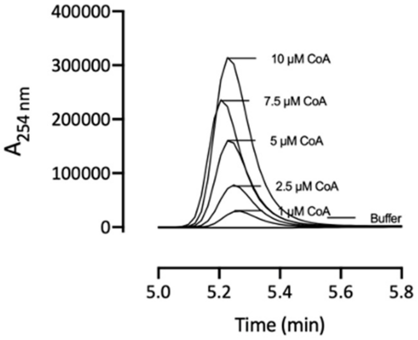

Figure S4. HPLC chromatograms of CoA standards. HPLC chromatograms of CoA standards (1, 2.5, 5, 7.5 and 10 μM) are shown. CoA isocratic elution (~5.2 min) was monitored at $A_{254\ nm}$. The graph was generated using GraphPad Prism8 (version 8.3.1).

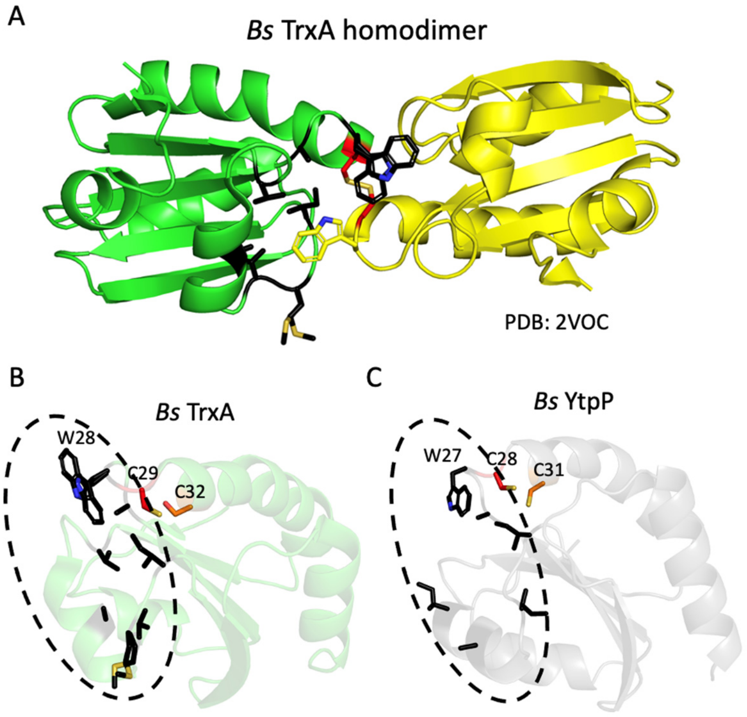

Figure S5. Structural analysis of *B. subtilis* TrxA and YtpP. (A) The structure of homodimeric *B. subtilis* TrxA (PDB: 2VOC), where the first subunit is colored in green and the second in yellow. The Cys29-Cys29 intermolecular disulfide bond is shown in red sticks, and the hydrophobic residues within the first subunit are shown in black sticks. Trp28 within the second subunit is shown in yellow sticks. The structures of *B. subtilis* TrxA **(B)** and YtpP (alpha fold) **(C)** are shown in green and gray Cartoon, respectively. The nucleophilic cysteine residues are shown in red and the resolving cysteines in orange. The residues forming a hydrophobic pocket (black dotted circle) near the nucleophilic cysteine are shown in black sticks.

Supplementary Tables

Table S1. CoA is released during deCoAlation of CoA-MsrA and CoA-PRDX5. The average CoA peak areas and the concentrations of CoA released following deCoAlation are shown. Each CoAlated substrate (CoA-MsrA and CoA-PRDX5) is either incubated with DTT, YtpP or TrxA.

	Average peak area	Average concentration (μM)
CoA-MsrA +DTT	2199974	7.6
CoA-MsrA +YtpP	2111366	7.3
CoA-MsrA +TrxA	2217392	7.6
CoA-PRDX5 +DTT	3794488	13.1
CoA-PRDX5 +YtpP	4378674	15.1
CoA-PRDX5 +TrxA	4398316	15.2
